# Aqueous Concentrations of Vascular Endothelial Growth Factor in Eyes with High Myopia with and without Choroidal Neovascularization

**DOI:** 10.1155/2013/257381

**Published:** 2013-03-06

**Authors:** Taku Wakabayashi, Yasushi Ikuno, Yusuke Oshima, Toshimitsu Hamasaki, Kohji Nishida

**Affiliations:** ^1^Department of Ophthalmology, Osaka University Graduate School of Medicine, 2-2 Yamadaoka, Suita, Osaka 565-0871, Japan; ^2^Department of Biomedical statistics, Osaka University Graduate School of Medicine, Osaka 565-0871, Japan

## Abstract

*Purpose*. To investigate aqueous concentrations of vascular endothelial growth factor (VEGF) in eyes with myopic choroidal neovascularization (CNV). *Methods*. Aqueous samples were collected, and VEGF concentrations were measured by enzyme-linked immunosorbent assay in 16 eyes (16 patients) with active myopic CNV, 23 eyes (16 patients) with high myopia without myopic CNV, and 8 control eyes (7 patients). Differences in the concentrations of VEGF in each group were compared. *Results*. The estimated mean VEGF concentrations were significantly lower in eyes with myopic CNV (82.0 pg/mL) (*P* = 0.016 ) and with high myopia without myopic CNV (58.9 pg/mL) (*P* < 0.001) compared with controls (116.6 pg/mL). The estimated mean VEGF concentration was significantly (*P* < 0.05) higher in eyes with myopic CNV than in those without myopic CNV in highly myopic eyes. In eyes with high myopia with and without CNV, the VEGF concentration was significantly (stepwise regression analysis, *R* = 0.325, *P* = 0.044) associated with the presence of myopic CNV but not with age, axial length, or intraocular pressure. *Conclusion*. Increased levels of VEGF may play a role in the pathogenesis of CNV in highly myopic eyes.

## 1. Introduction

High myopia is a major cause of blindness in Asia and Europe and in some ethnic groups in the United States [1–4]. Choroidal neovascularization (CNV) is a major cause of high myopia-related vision loss that occurs in 4% to 11% of patients with high myopia, with most eyes progressing to 20/200 or worse within 5 to 10 years after onset [5].

The pathogenesis of myopic CNV is unclear. However, vascular endothelial growth factor (VEGF), an angiogenic and vasopermeability factor, has been suggested to play a potential role in myopic CNV. The histologic and immunohistochemical studies of surgically excised myopic CNV membranes indicated that VEGF expression is high in the CNV membrane and may be involved in the development of myopic CNV [6]. A clinical association of myopic CNV with an increased aqueous humor level of VEGF has been reported [7]. Based on the histologic and clinical studies, intravitreal injection of anti-VEGF agents directly targeting VEGF has been introduced as a therapeutic strategy for myopic CNV and shown to stabilize the CNV activity and improve vision [8–13].

Although previous studies strongly support the involvement of VEGF in myopic CNV, a recent study reported that aqueous humor levels of VEGF are much lower than previously reported [14] in eyes with myopic CNV, which is inconsistent with previous reports [7]. Therefore, it is still unclear if the intraocular concentration of VEGF in patients with myopic CNV is elevated substantially. We conducted the current study to investigate the aqueous concentrations of VEGF in eyes with and without CNV in eyes with high myopia and to assess a correlation between VEGF levels and several clinical parameters.

## 2. Patients and Methods

### 2.1. Patients

A consecutive series of patients with pathologic myopia who visited the Department of Ophthalmology, Osaka University Medical School Hospital, for treatment of newly developed, active myopic CNV or for cataract surgery between September 2008 and January 2009 was offered enrollment in this study. Patients with a history of vitreous surgeries for macular holes or myopic foveoschisis or other treatments, such as photodynamic therapy or photocoagulation, for previous CNV lesions were excluded. Pathologic myopia was defined as a spherical equivalent refractive error of −6.0 diopters or greater. The presence of myopic CNV was determined using fluorescein angiography (FA) and the agreement of two authors (TW and YI). Sixteen eyes with active myopic CNV and 23 eyes with high myopia without myopic CNV were ultimately included. The control group included eight eyes with a cataract or an epiretinal membrane (ERM) without diabetes mellitus or any other ocular diseases. Those patients were scheduled for cataract surgery or pars plana vitrectomy. All participants provided informed consent, and all agreed to the collection and analysis of samples. The study was performed according to the tenets of the Declaration of Helsinki. The off-label use of bevacizumab (Avastin, Roche, Basel, Switzerland) to treat myopic CNV and collection of aqueous humor samples for analysis was approved by the Institutional Review Board of Osaka University Medical School Hospital, Suita, Japan.

### 2.2. Ophthalmic Examination

All patients underwent a comprehensive ophthalmologic examination, including measurements of the best-corrected visual acuity (BCVA) and intraocular pressure (IOP), binocular indirect ophthalmoscopy, contact lens slit-lamp biomicroscopy, spectral-domain optical coherence tomography (Cirrus HD-OCT, Carl Zeiss Meditec, Dublin, CA, USA), and fundus photography. FA was performed in patients with myopic CNV. The retinal and choroidal thicknesses at the fovea also were measured on Cirrus HD-OCT in patients with myopic CNV. The medical records of the patients were reviewed for age, sex, axial length, IOP, lens status, central retinal thickness, and choroidal thickness. The retinal and choroidal thicknesses were measured using OCT, as previously described [15].

### 2.3. Sample Collection and Measurement of Vascular Endothelial Growth Factor Concentrations

Intravitreal injection of bevacizumab was performed in eyes with myopic CNV according to a strict aseptic protocol [16]. Briefly, topical anesthesia with 4% lidocaine and conjunctival disinfection with 1.25% povidone iodine solution was applied, followed by scrubbing of the eyelids and lashes with 10% povidone iodine. Bevacizumab (1 mg/40 *μ*L) was injected into the vitreous cavity via the pars plana.

A 200-*μ*L sample of undiluted aqueous humor was obtained using a 30-gauge needle immediately before injection of bevacizumab or surgery in myopic patients. Undiluted aqueous humor samples (200 *μ*L) also were obtained immediately before surgery from patients with a cataract or an ERM without high myopia as control samples. All samples were collected in sterile syringes and immediately transferred to dry ice and stored at −80°C until the time of assay.

Aqueous humor levels of VEGF were measured by an enzyme-linked immunosorbent assay using a kit (Quantikine, Human VEGF immunoassay, R&D System Inc., Minneapolis, MN, USA) for human VEGF. The primary antibody against VEGF detected two major soluble forms (VEGF_121_ and VEGF_165_) of the four VEGF isoforms. An experienced technician (Mitsubishi Chemical Evidence Inc., Tokyo, Japan), who was masked to the samples' details, performed the assays. The lower detectable limit of the VEGF concentration was 15.6 pg/mL.

### 2.4. Statistical Analysis

The baseline characteristics are expressed as the mean ± standard deviation (SD) for continuous variables and frequencies for categorical variables. Comparisons of the baseline characteristics were performed by one-way analysis of variance (ANOVA) for continuous variables and chi-square tests for categorical variables. Analysis of possible predictors of VEGF concentrations was done using multiple linear regression analysis. However, because several values of VEGF concentrations were left censored, the regression technique for left-censored data using the quasi-EM algorithm method was used for the analysis [17, 18]. The predictors were included and excluded using the stepwise method. The stepwise regression for left-censored data was done with the validated in-house Fortran program. All other analyses were performed using SAS software, version 9.1 (SAS Institute, Cary, NC, USA). All *P* values were calculated from two-tailed tests; *P* values less than 0.05 were considered statistically significant.

## 3. Results 

A total of 47 eyes (16 with myopic CNV; 23 with high myopia without myopic CNV; eight with a cataract or an ERM without high myopia) of 39 patients were included in this study. The mean patient age was 65.2 ± 8.5 years (range, 51–82 years). The patient demographics and baseline characteristics are shown in Table 1. There were no significant differences in age and gender among the 16 eyes with myopic CNV, 23 eyes with high myopia and without myopic CNV, and eight eyes with a cataract or ERM without high myopia. 

**Figure 1:**
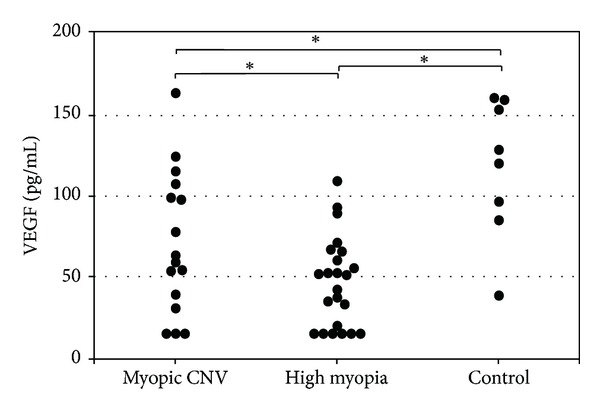
Vascular endothelial growth factor concentrations in the aqueous humor in eyes with myopic choroidal neovascularization (CNV), high myopia without myopic CNV, and controls. *P < 0.05.

The estimated mean concentrations of VEGF in the aqueous humor of the 16 eyes with myopic CNV and 23 eyes with high myopia and without myopic CNV were 82.0 pg/mL (95% confidence interval (CI), 65.4–98.7) and 58.9 pg/mL (95% CI, 44.2–73.6), respectively (Figure 1). The estimated mean concentration of VEGF in the aqueous humor of the control group was 116.6 pg/mL (95% CI, 94.2–138.9). The estimated mean VEGF concentration was significantly lower in eyes with myopic CNV (*P* = 0.016) and high myopia without myopic CNV (*P* < 0.001) compared with controls. However, the estimated mean VEGF concentration was significantly (*P* = 0.041) higher in eyes with myopic CNV than without myopic CNV in highly myopic eyes. In eyes with high myopia with and without CNV, the VEGF concentration was significantly (stepwise regression analysis, *R* = 0.325, *P* = 0.044) associated with the presence of myopic CNV but not with age, axial length, or IOP. 

**Figure 2:**
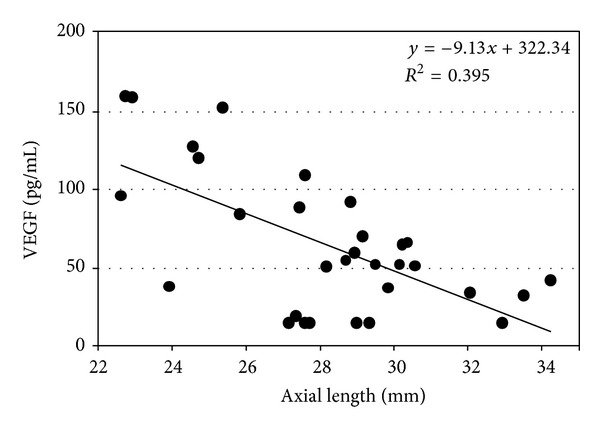
The graph shows the association between the vascular endothelial growth factor (VEGF) concentration in the aqueous humor and the axial length in eyes with high myopia without myopic choroidal neovascularization (CNV) and controls. The VEGF concentration is correlated negatively with the axial length (*R* = 0.629, *P* < 0.001).

In eyes without myopic CNV, that is, high myopia without myopic CNV and control eyes, the VEGF concentration was correlated negatively with the axial length (*R* = 0.629, *P* < 0.001) (Figure 2). However, the VEGF concentration was not associated with age, gender, IOP, age, or lens status in those groups.

In eyes with myopic CNV, the VEGF concentration was not associated with age, gender, IOP, axial length, central choroidal thickness, or CNV location or size (greatest linear diameter) (Table 2). The VEGF concentration tended to be associated with the central retinal thickness but the correlation did not reach statistical significance (*R* = 0.447, *P* = 0.083).

## 4. Discussion

VEGF is thought to play a major role in ocular neovascular diseases, such as diabetic retinopathy, CNV due to age-related macular degeneration, and neovascular glaucoma [18–23]. Aqueous humor levels of VEGF in patients with intraocular neovascularization are highly elevated and sufficient to stimulate human endothelial cell migration and proliferation [24]. Based on those previous studies, it is reasonable that the intraocular concentration of VEGF also may be elevated and related to the development of CNV associated with pathologic myopia. However, the role of VEGF in myopic CNV is unclear, because no comparative study has evaluated the intraocular VEGF concentration in eyes with and without CNV in patients with high myopia. 

In the current study, the physiologic expression of VEGF in the aqueous humor in highly myopic eyes was significantly lower than that in the control eyes and in previous reports with nonmyopic patients [18, 19, 22, 23]. The VEGF concentration was correlated negatively with the axial length. Therefore, the VEGF concentration may be diluted because of the large vitreous cavity due to the elongated axial length in highly myopic eyes. It is uncertain if the low aqueous levels of VEGF represent decreased production of VEGF from endothelial cells and retinal pigment epithelium (RPE) in highly myopic eyes.

Thinning and loss of the lamina of the small choroidal vessels and concomitant atrophic changes in the RPE are associated with myopic chorioretinal atrophy, resulting in subsequent visual impairment in pathologic myopia [25–30]. Those changes are attributed to the substantial axial elongation that results in stretching the scleral, choroidal, and retinal tissue. Therefore, we speculated that the low levels of VEGF may not contribute directly to the pathogenesis of chorioretinal atrophy in pathologic myopia. In addition, the choroidal thickness did not account for the VEGF levels in the current study. However, further studies are needed to elucidate if the low levels of VEGF promote further degeneration of the choroidal and retinal tissue in pathologic myopia. 

Although the aqueous humor levels of VEGF in eyes with myopic CNV were substantially lower than in the control eyes, the aqueous humor levels of VEGF were significantly higher compared with the levels in eyes without myopic CNV in highly myopic eyes. In addition, the VEGF concentration was significantly (*R* = 0.325, *P* = 0.044) associated with the presence of myopic CNV in eyes with high myopia, indicating the potential role of VEGF in the pathogenesis of myopic CNV. According to recent studies that evaluated the angiographic features in eyes with myopic CNV, a choroidal filling delay may be related to the pathogenesis of myopic CNV [27]. The choroidal circulatory disturbance caused by the disappearance of the choroidal vessels and/or capillaries in pathological myopia may induce hypoxia in the RPE and glial cells, which are an important source of VEGF, and subsequent upregulation of VEGF expression. Elevated levels of VEGF may trigger proliferation of choroidal vascular endothelial cells and induce development of CNV. Because the VEGF levels in the aqueous are correlated with those in the vitreous [31], elevated aqueous levels of VEGF may reflect the sequential events that occur in the posterior pole in eyes with myopic CNV. However, further investigation is needed to determine if the increased intraocular levels of VEGF expression actually induce CNV or simply result from the development of CNV.

The limitations of the current study were the relatively small number of eyes in each group, the inability to measure the VEGF levels below the lower limit of detection of 15.6 pg/mL, and the absence of data on the vitreous concentration of VEGF, which may be a greater reflection of the pathology of the choroid, the RPE, or the CNV lesions. 

In conclusion, the current study showed for the first time that the VEGF concentration in eyes with high myopia is significantly lower compared with control eyes and that in eyes with myopic CNV, the VEGF level is significantly elevated compared with physiologic levels in highly myopic eyes. Although we did not find an association between the VEGF levels and patient age, gender, axial length, IOP, central retinal thickness, or central choroidal thickness in eyes with myopic CNV because of the small number of eyes in this group, further studies with more patient data would elucidate the potential association between intraocular VEGF and CNV activity, or the underlying choroidal status leading to the development of CNV.

## Figures and Tables

**Table 1:** Characteristics of eyes with myopic choroidal neovascularization, high myopia without myopic choroidal neovascularization, and control.

Parameter	Total (*n* = 47)	mCNV (*n* = 16)	High myopia without mCNV (*n* = 23)	Control (*n* = 8)	*P* value
Number of eyes/patients	47/39	16/16	23/16	8/7	
Sex (F/M, number of patients)	24/15	9/7	11/5	4/3	0.74
Age (yrs)					
Mean ± SD	65.2 ± 8.5	65.5 ± 10.4	65.1 ± 7.9	64.6 ± 6.0	0.97
Range	51–82	51–82	51–77	54–71	
Axial length (mm)					
Mean ± SD	28.7 ± 2.8	29.7 ± 1.8	29.6 ± 2.0	24.1 ± 1.2	<0.001
Range	22.6–34.2	26.9–32.6	27.2–34.2	22.6–24.2	
Intraocular pressure (mmHg)					
Mean ± SD	15.5 ± 2.8	15.9 ± 2.8	15.1 ± 3.0	15.5 ± 1.9	0.677
Range	8–20	11–20	8–20	12–17	
Lens status					
Phakia	41	11	22	8	—
Pseudophakia	6	5	1	0	

mCNV: myopic choroidal neovascularization; F/M: female/male; SD: standard deviation.

**Table 2:** Clinical parameter of the eyes with myopic choroidal neovascularization.

Parameter	mCNV (*n* = 16)
Central retinal thickness	
Mean ± SD	346 ± 150
Range	52–564
Choroidal thickness (*µ*m)	
Mean ± SD	64 ± 17
Range	32–94
CNV location, *n* (%)	
Subfoveal	10
Juxtafoveal	3
Extrafoveal	3
CNV size (GLD; *μ*m)	
Mean ± SD	1524 ± 654
Range	547–2528

GLD: greatest linear diameter; logMAR: logarithm of the minimum angle of resolution; mCNV: myopic choroidal neovascularization; SD: standard deviation.
